# Genotypic Males Play an Important Role in the Creation of Genetic Diversity in Gynogenetic Gibel Carp

**DOI:** 10.3389/fgene.2021.691923

**Published:** 2021-05-28

**Authors:** Xin Zhao, Zhi Li, Miao Ding, Tao Wang, Ming-Tao Wang, Chun Miao, Wen-Xuan Du, Xiao-Juan Zhang, Yang Wang, Zhong-Wei Wang, Li Zhou, Xi-Yin Li, Jian-Fang Gui

**Affiliations:** ^1^State Key Laboratory of Freshwater Ecology and Biotechnology, Institute of Hydrobiology, The Innovative Academy of Seed Design, Hubei Hongshan Laboratory, Chinese Academy of Sciences, Wuhan, China; ^2^College of Life Sciences, University of Chinese Academy of Sciences, Beijing, China

**Keywords:** unisexual reproduction, gynogenesis, genetic diversity, Muller’s ratchet, genotypic sex determination, temperature-dependent sex determination

## Abstract

Unisexual lineages are commonly considered to be short-lived in the evolutionary process as accumulation of deleterious mutations stated by Muller’s ratchet. However, the gynogenetic hexaploid gibel carp (*Carassius gibelio*) with existence over 0.5 million years has wider ecological distribution and higher genetic diversity than its sexual progenitors, which provides an ideal model to investigate the underlying mechanisms on countering Muller’s ratchet in unisexual taxa. Unlike other unisexual lineages, the wild populations of gibel carp contain rare and variable proportions of males (1–26%), which are determined via two strategies including genotypic sex determination and temperature-dependent sex determination. Here, we used a maternal gibel carp from strain F to be mated with a genotypic male from strain A^+^, a temperature-dependent male from strain A^+^, and a male from another species common carp (*Cyprinus carpio*), respectively. When the maternal individual was mated with the genotypic male, a variant of gynogenesis was initiated, along with male occurrence, accumulation of microchromosomes, and creation of genetic diversity in the offspring. When the maternal individual was mated with the temperature-dependent male and common carp, typical gynogenesis was initiated that all the offspring showed the same genetic information as the maternal individual. Subsequently, we found out that the genotypic male nucleus swelled and contacted with the female nucleus after fertilization although it was extruded from the female nucleus eventually, which might be associated with the genetic variation in the offspring. These results reveal that genotypic males play an important role in the creation of genetic diversity in gynogenetic gibel carp, which provides insights into the evolution of unisexual reproduction.

## Introduction

Compared with the prevalence in unicellular eukaryotes, plants and invertebrates (Roach et al., 2014), unisexual reproduction is rare in vertebrates (Avise, 2015; Burke and Bonduriansky, 2017; Li and Gui, 2018). Since Amazon molly (*Poecilia formosa*) was first described to reproduce unisexually (Hubbs and Hubbs, 1932), about 100 taxa of vertebrates have been revealed (Avise, 2015) to have the unisexual reproductive ability via three strategies including parthenogenesis (Lampert and Schartl, 2010), gynogenesis (Gui and Zhou, 2010), and hybridogenesis (Lavanchy and Schwander, 2019). Unisexual vertebrates generate all-female offspring with nearly identical genetic information, which is predicted to have many disadvantages in the evolutionary process. Commonly, unisexual reproduction without meiosis and meiotic recombination is supposed to accumulate deleterious mutation (Muller’ ratchet) (Muller, 1964; Neaves and Baumann, 2011; Warren et al., 2018) and hinder the creation of genetic diversity (Van Valen, 1973; Strotz et al., 2018; Warren et al., 2018), which will lead to the eventual extinction. However, some unisexual taxa, such as Amazon molly (*P. formosa*) (Loewe and Lamatsch, 2008; Warren et al., 2018), salamanders (*Ambystoma*) (Bi and Bogart, 2010; Bogart, 2019), and gibel carp (*Carassius gibelio*) (Liu et al., 2017a, b), have wider ecological distribution than their relative sexual progenitors and have outlived their predicted time of extinction. Illustration of the underlying mechanisms how these unisexual lineages adapt to changing environment and live for a long time will provide insights on the evolution of reproduction modes.

Hexaploid gibel carp (*C. gibelio*) was originated from sympatric progenitor tetraploid crucian carp (*Carassius auratus*) via autotriploidy about 0.5 million years ago (Mya) (Li et al., 2014). Genomic anatomy and single-gene analysis revealed that gibel carp was an allohexaploid (AAABBB) in comparison with the allotetraploid crucian carp (AABB) (Li et al., 2014; Gan et al., 2021; Yu et al., 2021; Wang et al., under review). Three alleles in subgenome A and three alleles in subgenome B make the genome unable to pair during meiosis (Gan et al., 2021) and the hexaploid gibel carp conquers the reproductive obstacles via unisexual gynogenesis (Gui and Zhou, 2010; Zhou and Gui, 2017), in which unreduced eggs are activated by the sperm of sympatric sexual species to initiate embryogenesis with only maternal genetic information. Compared with sexual tetraploid crucian carp, gynogenetic hexaploid gibel carp exhibits wider geographic distribution and higher genetic diversity (Jiang et al., 2013; Liu et al., 2017a). However, how the genetic variation occurs in hexaploid gibel carp with unisexual gynogenesis remains elusive.

Although hexaploid gibel carp reproduce through unisexual gynogenesis, rare and variable proportions of males (1–26%) have been observed in natural populations of hexaploid gibel carp (Jiang et al., 2013; Li et al., 2018). These males are revealed to be determined via genotypic sex determination (GSD) or environmental sex determination (ESD) (Mei and Gui, 2015; Li and Gui, 2018), in which supernumerary microchromosomes and larval rearing temperature are the key determinants, respectively (Li et al., 2016, 2018; Li and Gui, 2018). Here we used a maternal individual from strain F of gibel carp to be mated with the genotypic and temperature-dependent male from strain A^+^, and then investigated the sex ratio, microchromosome number, and genetic diversity in the offspring. Male occurrence, microchromosome accumulation, and genetic variation were observed only in the offspring derived from the genotypic male, which might be associated with the swelling of paternal nucleus after fertilization. These findings revealed that genotypic males played an important role in the creation of genetic diversity in gynogenetic gibel carp, which shed light on the environmental adaption and long existence in unisexual vertebrates.

## Materials and Methods

### Experimental Fish Source

All experimental fish including gibel carp and common carp were obtained from the National Aquatic Biological Resource Center (NABRC), Institute of Hydrobiology, Chinese Academy of Sciences, Wuhan, China. Strain A^+^ (Wang et al., 2011), strain F (Chen et al., 2020) of hexaploid gibel carp, and red common carp were used in this study. Genotypic males of strain A^+^ were obtained from the offspring of the sexual reproductive family, which were reared under normal temperature (about 20°C) during larval period for 30 days (Zhu et al., 2018). Temperature-dependent males of Strain A^+^ were obtained from the offspring of the unisexual gynogenetic family, which were reared under high rearing temperature treatment (about 32°C) during larval period for 30 days as previously described (Zhu et al., 2018).

### Artificial Propagation and Fish Culture

During the propagation season of gibel carp, the maternal individual from strain F was induced into ovulation by intraperitoneal injection with a mixture of acetone-dried carp pituitary, human chorionic gonadotropin, and luteinizing hormone releasing hormone as previously described (Sun et al., 2010). About 8 h after injection, the maternal individual began to spawn and was mated with a genotypic male from strain A^+^, a temperature-dependent male from strain A^+^, and a male from another species common carp, respectively. All the progenies were reared at 20°C (±1°C) for 30 days during the larval period and then were maintained in outdoor tanks.

### Genomic DNA Extraction and PCR Detection

Genomic DNA was extracted from a small piece of fin for each sampled fish as previously described (Wang et al., 2021). The primer pair *Cg*-MSM-F and *Cg*-MSM-R developed previously (Supplementary Table 1; Li et al., 2016) were used to detect the genotypic male-specific marker via 20 μl Taq DNA Polymerase reaction mix using TsingKe^®^ Master Mix (TsingKe, China). PCR amplification was performed on thermocycler ETC811 (EastWin, China). PCR cycling conditions were: 94°C for 4 min; 35 cycles of 94°C for 30 s, 58°C for 30 s, 72°C for 30 s; 72°C for 5 min; endless 4°C. And PCR amplification products were detected by 1% agarose gel electrophoresis.

### Chromosome Preparation and Fluorescence *in situ* Hybridization

Chromosome preparations were acquired through the method of head-kidney cell-phytohemagglutinin culture *in vivo* as described previously (Zhu et al., 2006). Fluorescence *in situ* hybridization was performed according to the method described previously with some modifications (Li et al., 2016). The slides with chromosome metaphase spreads were first treated at 60°C for 2 h and then denatured in 70% deionized formamide in 2× SSC for 3 min at 53°C; dehydrated in a precooled 70%, 90%, and 100% ethanol series for 5 min each; and lastly air-dried. A 60 ml hybridization mixture containing 100 ng labeled probe, 50% deionized formamide, 20% dextran sulfate, 0.5 mg/ml sheared salmon sperm DNA, 0.1% SDS, and 2× SSC was denatured in 73°C for 5 min and then put in ice immediately. The denatured mixture was dropped on the slides and covered with a coverslip. Next, hybridization was carried out in a moist chamber at 37°C for 12 h. The slides were washed on the shaker for 5∼10 min in each of the following: 2× SSC with 20% deionized formamide, 2× SSC with 0.1% Tween 20, 0.1× SSC with 0.1% Tween 20, 1× PBS with 0.1% Tween 20, and 1× PBS. Lastly, the slides were stained with 0.25 mg/ml DAPI (Sigma, United States) in 1× PBS solution, and then the images were observed and acquired by Zeiss Axio Imager 2 (Carl Zeiss, Germany) as previously described (Lu et al., 2021). The number of microchromosomes in each tested fish was obtained according to the most frequent number by counting fifty metaphases. The microchromosome probes (Sangon Biotech, China) were designed according to a repetitive satellite sequence and labeled with Cy3 (Ding et al., under review).

### Microsatellite Analysis

Microsatellite DNA, also known as simple sequence repeats (SSR), consists of 1–6 base pairs of core sequences repeated in tandem, which has a random distribution throughout the entire genome (Zhou et al., 2001). In this study, fifteen pairs of microsatellite primers (Supplementary Table 2; Zhou et al., 2001; Guo and Gui, 2008) that have been proved to be able to distinguish between strain F and strain A^+^ in gibel carp were used to explore the genetic contribution of the paternal individuals. PCR amplification was performed on thermocycler ETC811 (EastWin, China) in 20 μl Taq DNA Polymerase reaction mix using TsingKe^®^ Master Mix (TsingKe, China). PCR cycling conditions were performed as previously described (Zhou et al., 2001; Guo and Gui, 2008). PCR products were electrophoresed on 8% polyacrylamide gel and stained with GelRed (Biosharp, China). The target microsatellite bands were obtained using Poly-Gel DNA Extraction Kit (Solarbio, China) as instructions described and transformed into the PMD18-T vector (Takara, Japan) for sequencing. 10 positive clones were sequenced for each sample and the sequences were analyzed by the DNAMAN software for homologous analysis.

### DAPI Staining of Nuclear Behavior in the Fertilized Eggs

The ovulated eggs of the maternal individual from strain F were inseminated by sperm of a genotypic male from strain A^+^, a temperature-dependent male from strain A^+^, and a male common carp, respectively. The fertilized eggs were digested by 0.25% trypsin to remove their shells and then incubated at 24°C. The fertilized eggs at different developmental stages were fixed with 4% paraformaldehyde in PBS at 4°C overnight on a working shaker. About 40 inseminated eggs were fixed at each time point as previously described (Zhang et al., 2015). After washing with PBS three times, the nuclei were stained using DAPI (Sigma, United States), and the images were obtained via confocal microscopy (NOL-LSM 710 Carl Zeiss) as described (Zhu et al., 2018).

## Results

### Genotypic Male Generates Male Offspring

**FIGURE 1:**
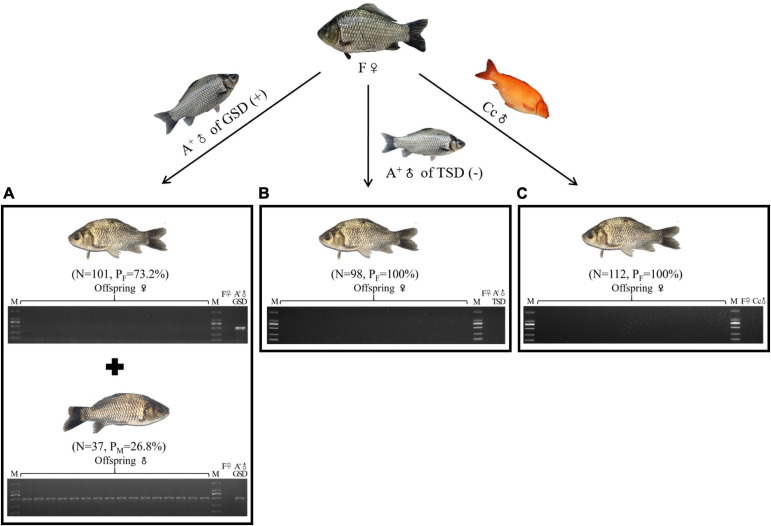
Male occurrence in the offspring. **(A–C)** Male occurrence in the offspring of F♀ mating with A^+^♂ of GSD **(A)**, A^+^♂ of TSD **(B)**, and Cc♂ **(C)**, respectively. PCR detection of GMM in the 16 randomly picked offspring and the parental individuals was shown at the bottom. ♀, female; ♂, male; (+), with GMM; (–), without GMM; N, number of female or male offspring; P_*F*_, female proportion; P_*M*_, male proportion; M, DL2000 marker.

To elucidate the genetic contributions of male individuals in gynogenetic gibel carp, we firstly used the same maternal fish from strain F (F♀) to be mated with a genotypic male from strain A^+^ (A^+^♂ of GSD), a temperature-dependent male from strain A^+^ (A^+^♂ of TSD), and a male from another species common carp (Cc♂) (Figure 1). In the family derived from A^+^♂ of GSD, 73.2% female and 26.8% male offspring were observed and the genotypic male-specific marker (GMM) (Li et al., 2016) was present in all sampled male offspring and the paternal A^+^♂ of GSD, which was absent in all female offspring and maternal F♀ (Figure 1A). However, the offspring derived from A^+^♂ of TSD was composed of all females and the GMM was absent in all the female offspring and parental individuals (Figure 1B), which was the same as the typical gynogenetic family stimulated by Cc♂ (Figure 1C). These results indicate that only the genotypic male can generate male offspring and the GMM is inherited stably across generations.

### Genotypic Male Leads to Accumulation of Microchromosomes in the Offspring

**FIGURE 2:**
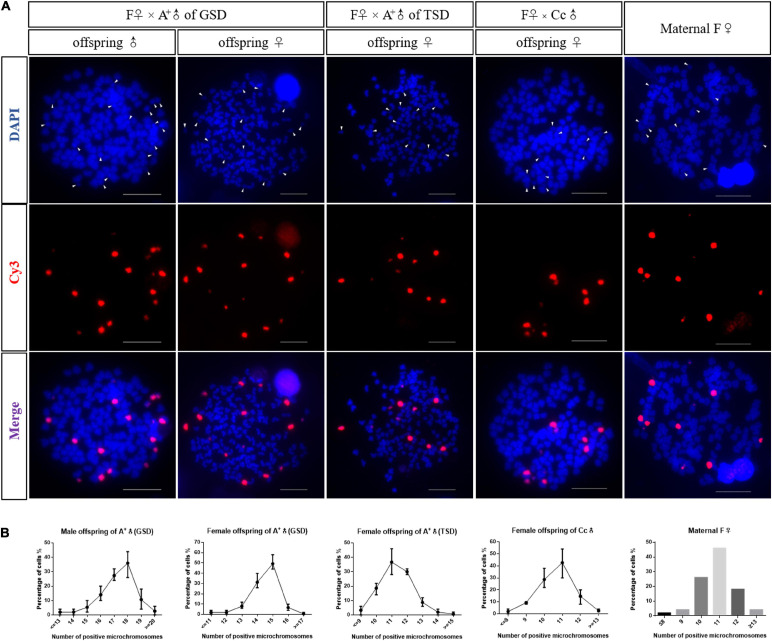
Microchromosome number in the offspring. **(A)** FISH analysis in metaphases of a male offspring of A^+^♂ of GSD, a female offspring of A^+^♂ of GSD, a female offspring of A^+^♂ of TSD, a female offspring of Cc♂, and the maternal F♀, respectively. Microchromosome probes were labeled with Cy3 and red fluorescence was produced. All metaphase chromosomes were stained with DAPI and appeared as blue. Arrowheads indicate microchromosomes. Scale bars represent 10 μm. **(B)** Statistical data of microchromosome number in male offspring of A^+^♂ of GSD, female offspring of A^+^♂ of GSD, female offspring of A^+^♂ of TSD, female offspring of Cc♂, and the maternal F♀. Three individuals were examined for each kind of offspring, and 50 metaphases were counted for each tested individual. Number of microchromosomes is exhibited on the *X*-axis, and the percentage of total cells is shown on the *Y*-axis. The data of microchromosome number in the maternal F♀ was represented in histogram. Detail statistical data of microchromosome number are given in Supplementary Figure 1.

Previous studies revealed that supernumerary microchromosomes played the genotypic male determination role in gibel carp (Li et al., 2016), so we analyzed the microchromosomes in offspring of the three families derived from F♀ mating with A^+^♂ of GSD, A^+^♂ of TSD, and Cc♂ (Figure 2). Fifty metaphases were measured for each individual and the most frequent microchromosome number was defined as the microchromosome number of this individual. In the family of F♀ mating with A^+^♂ of GSD, the most frequent microchromosome number of three male and three female offspring were 18 and 15, respectively, which indicated that male and female offspring had 18 and 15 microchromosomes, respectively (Figure 2 and Supplementary Figure 1). In the families of F♀ mating with A^+^♂ of TSD and Cc♂, the female offspring contained 11 microchromosomes, which had the same microchromosome number as the maternal F♀ (Figure 2 and Supplementary Figure 1). The microchromosomes in the offspring of F♀ mating with A^+^♂ of GSD were more than that in the offspring of the other two families and the common maternal F♀, indicating that the extra microchromosomes might be inherited from the paternal A^+^♂ of GSD. These findings imply that the genotypic male is able to pass on their microchromosomes to the next generation.

### Genotypic Male Contributes to Genetic Variation in the Offspring

**FIGURE 3:**
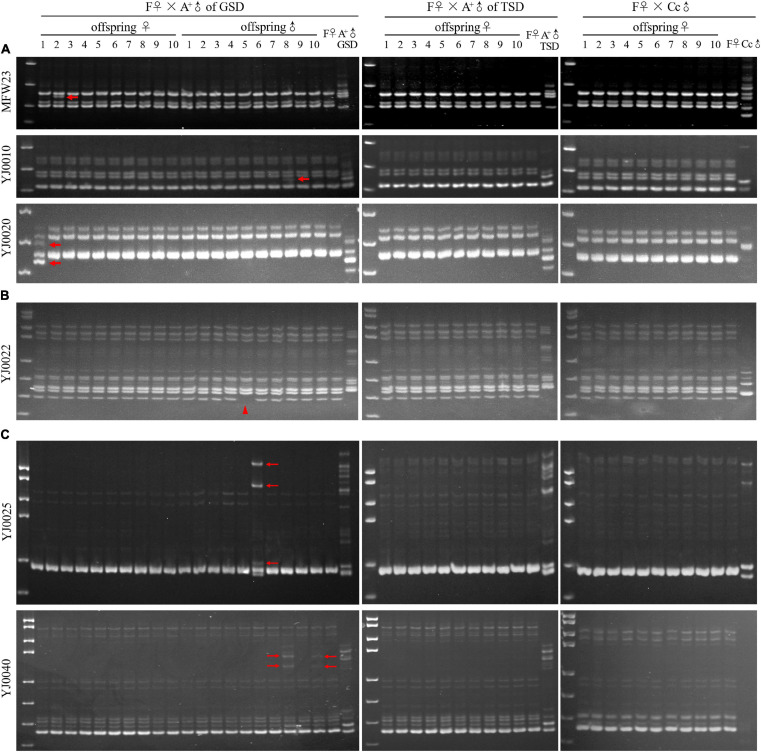
Microsatellite analysis in the families of F♀ mating with A^+^♂ of GSD, A^+^♂ of TSD, and Cc♂. **(A)** Microsatellite electrophoretic patterns amplified by the primer MFW23, YJ0010, and YJ0020. Thick arrows indicate offspring bands, which are inherited from paternal A^+^♂ of GSD. **(B)** Microsatellite electrophoretic patterns amplified by the primer YJ0022. The arrowhead indicates the clearance of maternal electrophoretic band. **(C)** Microsatellite electrophoretic patterns amplified by the primer YJ0025 and YJ0040. Thin arrows indicate the novel electrophoretic bands in the offspring, which are absent in parental individuals. ♀, female; ♂, male. Marker is pUC18 DNA/*Msp*I.

Subsequently, we performed microsatellite analysis to detect the genetic contribution of paternal males using 15 primer pairs (Supplementary Table 2; Zhou et al., 2001; Guo and Gui, 2008), which could produce different electrophoretic patterns between strain F and strain A^+^. In the family of F♀ mating with A^+^♂ of GSD, nine primer pairs generated the same electrophoretic patterns in all the examined offspring and the maternal F♀ (Supplementary Figure 2), while the other six primer pairs generated some variations of electrophoretic bands in a few offspring (Figure 3). These variations were divided into three categories, including inheritance of paternal electrophoretic bands (Figure 3A), absence of maternal electrophoretic bands (Figure 3B), and occurrence of novel electrophoretic bands (Figure 3C). The inheritance of paternal DNA was also verified via sequencing of the electrophoretic bands in both paternal individual and corresponding offspring (Supplementary Figure 3). In the families of F♀ mating with A^+^♂ of TSD and Cc♂, all the examined offspring displayed the same electrophoretic patterns as the maternal F♀ via using all the 15 primer pairs (Figure 3 and Supplementary Figure 2). Based on these results, we can conclude that when the maternal F♀ mating with A^+^♂ of TSD and Cc♂, typical gynogenesis was initiated and all the offspring have the same genetic information as the maternal individual. When the maternal F♀ mating with A^+^♂ of GSD, a gynogenetic variant was initiated that a few offspring presented some genetic variation compared with their maternal individual, although most offspring showed the same genetic information with their maternal individual.

### Sperm Nucleus Swelling of the Genotypic Male During Fertilization

**FIGURE 4:**
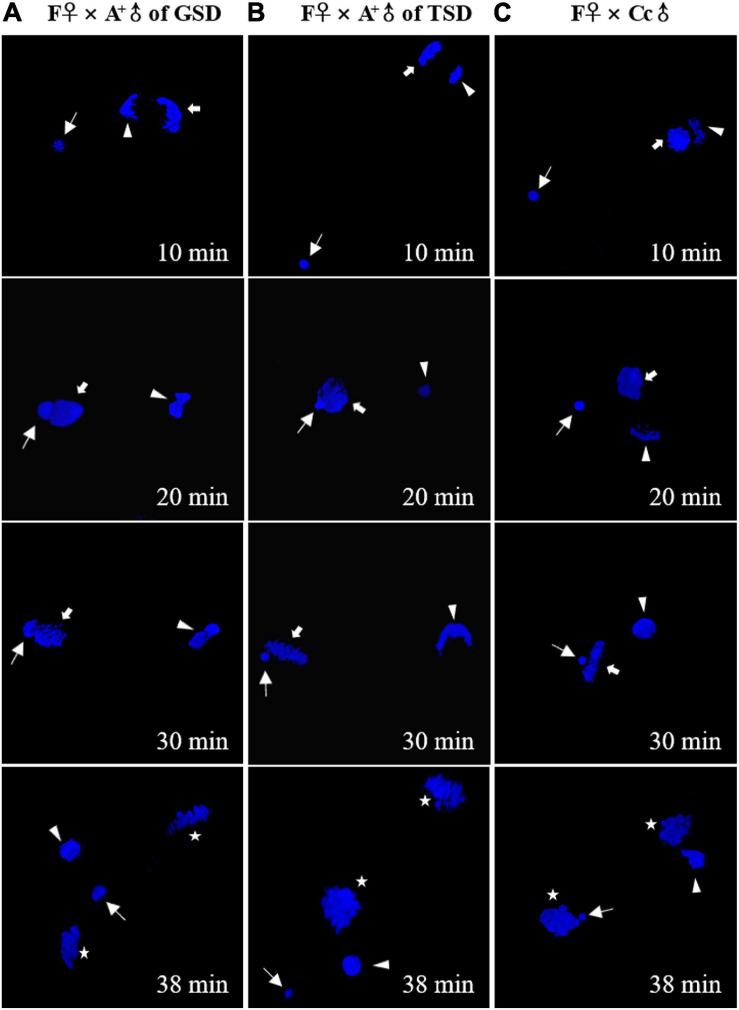
Nuclear behaviors in the fertilized eggs stained by DAPI. **(A–C)** The fertilized eggs of F♀ mating with A^+^♂ of GSD **(A)**, A^+^♂ of TSD **(B)**, and Cc♂ **(C)**. Thin arrows indicate sperm nucleus. Thick arrows indicate female pronucleus. Arrowheads indicate second polar-body. Asterisks indicate the nucleus of zygote after the first mitosis. The female pronucleus and the second polar-body are distinguished by the morphology. The corresponding time after fertilization is showed at the right corner.

To find out how the genetic diversity occurred in the offspring, we traced the nuclear behaviors in the fertilized eggs (Figure 4). When the eggs of F♀ were fertilized by the sperm from A^+^♂ of GSD, the sperm nucleus swelled and moved to the female pronucleus and contacted with the female pronucleus. However, the sperm nucleus was extruded from the female nucleus at last, and the female nucleus seemed to enter first mitosis by itself (Figure 4A). When the eggs of F♀ were fertilized by the sperm from A^+^♂ of TSD, the male nucleus stayed in a condensed status throughout the whole process after fertilization (Figure 4B), which was identical to the typical gynogenesis initiated by the sperm from Cc♂ (Figure 4C). Although the male nucleus from A^+^♂ of GSD was extruded from the female nucleus eventually, it swelled and contacted with the female nucleus from 20 min to 30 min after fertilization. This process might provide chances for introgression of paternal genetic information, clearance of some maternal genetic information, and creation of novel genetic information.

## Discussion

Although unisexual reproduction can avoid the mating costs and achieve high fecundity, its disadvantages are obvious that deleterious mutations cannot be purged and new genotypes cannot be created without meiosis (Muller, 1964; Wang et al., 2018; Warren et al., 2018). As clearance of deleterious mutations and creation of genetic diversity are essential for adaptation to changing environment, the unisexual vertebrates are predicted to be an evolutionary dead-end (Van Valen, 1973; Strotz et al., 2018; Warren et al., 2018). However, some unisexual taxa have lived much older than predicted and have wider ecological distributions than their sexual progenitors (Loewe and Lamatsch, 2008; Bi and Bogart, 2010; Liu et al., 2017a, b; Warren et al., 2018; Bogart, 2019). The hexaploid gibel carp was originated from ancestral tetraploid crucian carp via autotriploidy about 0.5 Mya (Li et al., 2014). Given a generation time of 1–2 years, hexaploid gibel carp has existed for about 250,000–500,000 generations and has exceeded 100,000 generations, which was the predicted extinction generation of a strict unisexual reproduction population (Lynch and Gabriel, 1990; Warren et al., 2018). Moreover, the gynogenetic hexaploid gibel carp exhibited wider geographic distributions and higher genetic diversity than its sexual progenitor tetraploid crucian carp (Liu et al., 2017a).

Unlike other unisexual vertebrates, gibel carp males were observed in natural populations, although the proportions of males were very low and variable (Li et al., 2018). There were two kinds of males in gibel carp that were determined via two strategies including GSD and TSD (genotypic male and temperature-dependent male). In this study, we revealed the differences between these two males on the contribution to diversity creation. When the maternal gibel carp was mated with temperature-dependent males or male individuals from other species, a typical gynogenesis was stimulated according to the all-female composition in the offspring (Figure 1), the same microchromosome number with the maternal individual (Figure 2), genetic identity with the maternal individual (Figure 3), and condensed status of male nucleus after fertilization (Figure 4). When the maternal gibel carp was mated with genotypic males, a variant mode of gynogenesis occurred, accompanied by male occurrence in the offspring (Figure 1), accumulation of microchromosomes (Figure 2), variation of genetic diversity (Figure 3), and the swelling of male nucleus after fertilization (Figure 4). The genotypic males can increase genetic diversity in the offspring via introgression of paternal DNA, deletion of maternal DNA, and occurrence of novel genetic information. Although the creation of genetic diversity happens at a low frequency, it will counter Muller’s ratchet at a certain level, which may contribute to the environmental adaption and long existence of gibel carp.

There are some popular hypotheses for countering the accumulation of mutations in absence of sex. First, occasional sexual reproduction can allow recombination and purge deleterious mutations. And these individuals with facultative reproductive strategies (where individuals can switch between unisexual and sexual reproductions) can possess the advantages of both reproduction modes (Burke and Bonduriansky, 2017; Wang et al., 2018). Second, introgression of DNA into the unisexual lineage can compensate for deleterious mutations, which has been used to partially explain a substantial polymorphism and high heterozygosity in gynogenetic Amazon molly (Warren et al., 2018). Third, unisexual polyploids can reduce spontaneous mutations by gene conversion, in which one sequence is replaced by its homologous sequence (Soppa, 2011; Maciver, 2016). Fourth, large populations allow the fittest individuals to be selected and exist, even though there are many deleterious mutations (Maciver, 2016). In gynogenetic gibel carp, not only the creation of genetic diversity can be caused by genotypic males (Figure 3), but also a high rate of gene conversion has been detected in gynogenetic line (Wang et al., under review), which may both lead to purging deleterious mutations.

Previous studies have revealed that supernumerary microchromosomes play genotypic male determination role (Li et al., 2016), and the supernumerary microchromosomes may be the main driving force of male occurrence in gynogenetic gibel carp (Ding et al., under review). However, we still don’t know how these microchromosome originate and what is the mechanism underlying the creation of genetic diversity triggered by genotypic males. Thus, further studies on the reproduction modes and sex determination systems of gynogenetic gibel carp will contribute to unveil these puzzles.

## Conclusion

In this study, we used hexaploid gibel carp, which can reproduce via unisexual gynogenesis but also contained variable proportions of males in wild populations, to illustrate how unisexual taxa countered Muller’s ratchet. We found out that the temperature-dependent male initiated typical gynogenesis that all the offspring had the same genetic information as the maternal individual. However, the genotypic male triggered a variant of gynogenesis along with male occurrence, accumulation of microchromosomes, and creation of genetic diversity in the offspring, which might be associated with the swelling of male nucleus after fertilization. Thus, the genotypic males play an important role in the creation of genetic diversity and benefit for the evolutionary long existence and environmental adaptations of gynogenetic gibel carp, which provides insights into the evolution of unisexual reproduction.

## Data Availability Statement

The original contributions presented in the study are included in the article/Supplementary Material, further inquiries can be directed to the corresponding author/s.

## Ethics Statement

All the protocols of fish experiments in this research were reviewed and approved by the Animal Care and Use Committee of the Institute of Hydrobiology, Chinese Academy of Sciences.

## Author Contributions

J-FG and X-YL designed the study. XZ, ZL, MD, TW, M-TW, CM, W-XD, X-JZ, YW, Z-WW, and LZ performed the experimental work and data analysis. X-YL and XZ wrote the manuscript with input from all other authors. J-FG revised the manuscript. All authors contributed to the article and approved the submitted version.

## Conflict of Interest

The authors declare that the research was conducted in the absence of any commercial or financial relationships that could be construed as a potential conflict of interest.

## Supplementary Material

Supplementary Figure 1 Histogram analyses of microchromosome number. **(A,B)** Microchromosome number in three male offspring **(A)** and three female offspring **(B)** from F♀ mating with A^+^♂ of GSD. **(C)** Microchromosome number in three female offspring from F♀ mating with A^+^♂ of TSD. **(D)** Microchromosome number in three female offspring from F♀ mating with Cc♂. A total of 50 metaphases were counted for each tested individual. Number of microchromosomes is exhibited on the *X*-axis and the percentage of total cells is shown on the *Y*-axis.

Supplementary Figure 2 Microsatellite analysis in the families of F♀ mating with A^+^♂ of GSD, A^+^♂ of TSD, and Cc♂. Microsatellite electrophoretic patterns amplified by the primer MFW1, MFW19, YJ0001, YJ0002, YJ0004, YJ0005, YJ0033, YJ0039, and YJ0042. ♀: female; ♂: male. Marker is pUC18 DNA/*Msp*I.

Supplementary Figure 3 Sequence alignments of the electrophoretic bands with the same size in both paternal A^+^♂ of GSD and corresponding offspring in Figure 3A. **(A,B)** Alignment of sequences amplified by primer MFW23 **(A)** and primer YJ0010 **(B)** between paternal individual A^+^♂ of GSD and the corresponding individual in the offspring. **(C,D)** Alignment of long sequences **(C)** and short sequences **(D)** amplified by primer YJ0020 between the paternal individual A^+^♂ of GSD and the corresponding individual in the offspring. Primers were exhibited on the left side. The forward and reverse primer sequences are indicated in red and blue rectangles, respectively.

Supplementary Table 1 Primer sequences and annealing temperature of genotypic male-specific marker.

Supplementary Table 2 Sequences and annealing temperatures of fifteen microsatellite primers.
