# Supplementary figures and images for: Genotypic Males Play an Important Role in the Creation of Genetic Diversity in Gynogenetic Gibel Carp
Source: Front Genet. 2021 May 28;12:691923. doi: 10.3389/fgene.2021.691923 (PMC8194356; doi:10.3389/fgene.2021.691923)

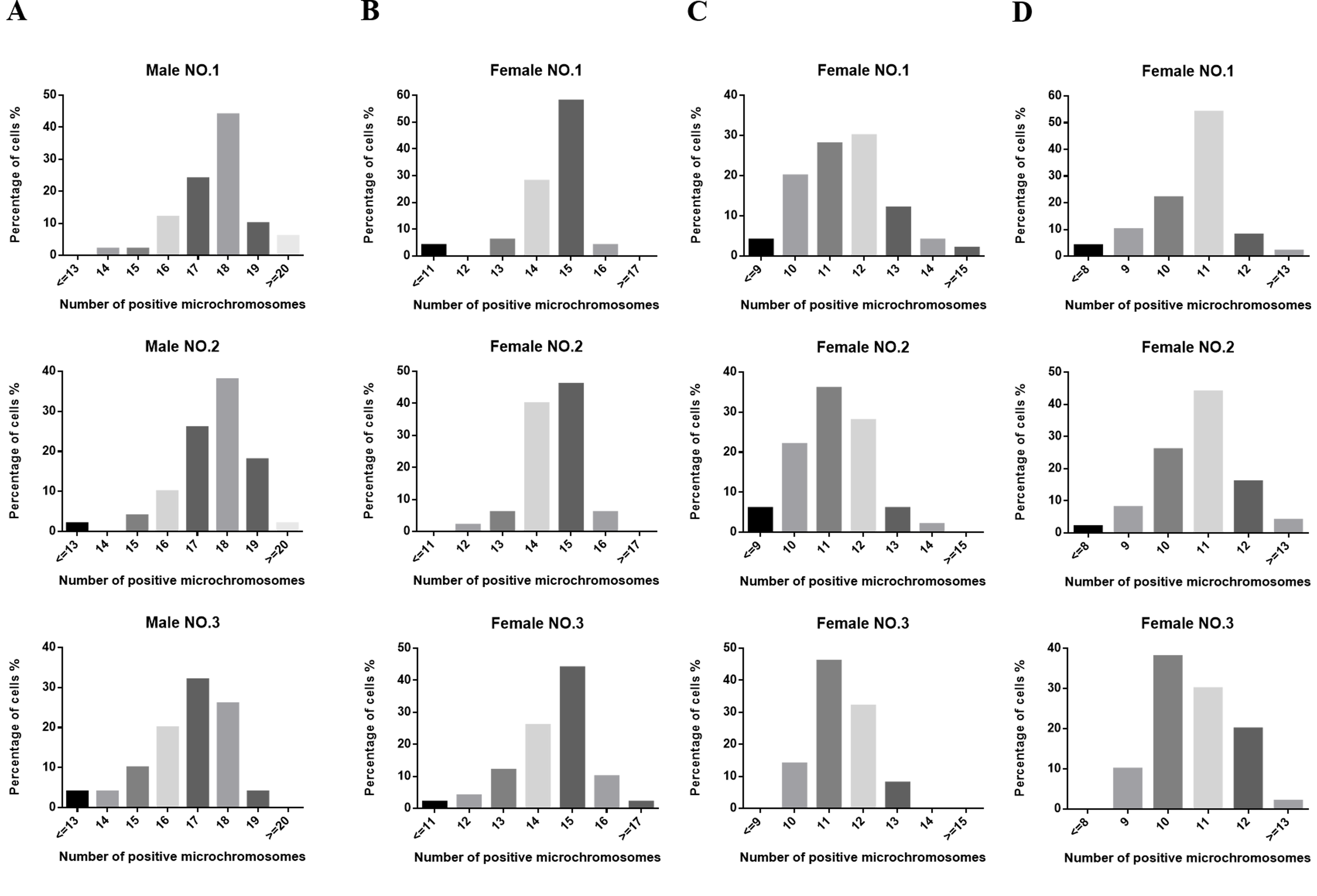

Supplement: Supplementary Figure 1 — Histogram analyses of microchromosome number. (A,B) Microchromosome number in three male offspring (A) and three female offspring (B) from F♀ mating with A+♂ of GSD. (C) Microchromosome number in three female offspring from F♀ mating with A+♂ of TSD. (D) Microchromosome number in three female offspring from F♀ mating with Cc♂. A total of 50 metaphases were counted for each tested individual. Number of microchromosomes is exhibited on the X-axis and the percentage of total cells is shown on the Y-axis. [file Image_1.tif]

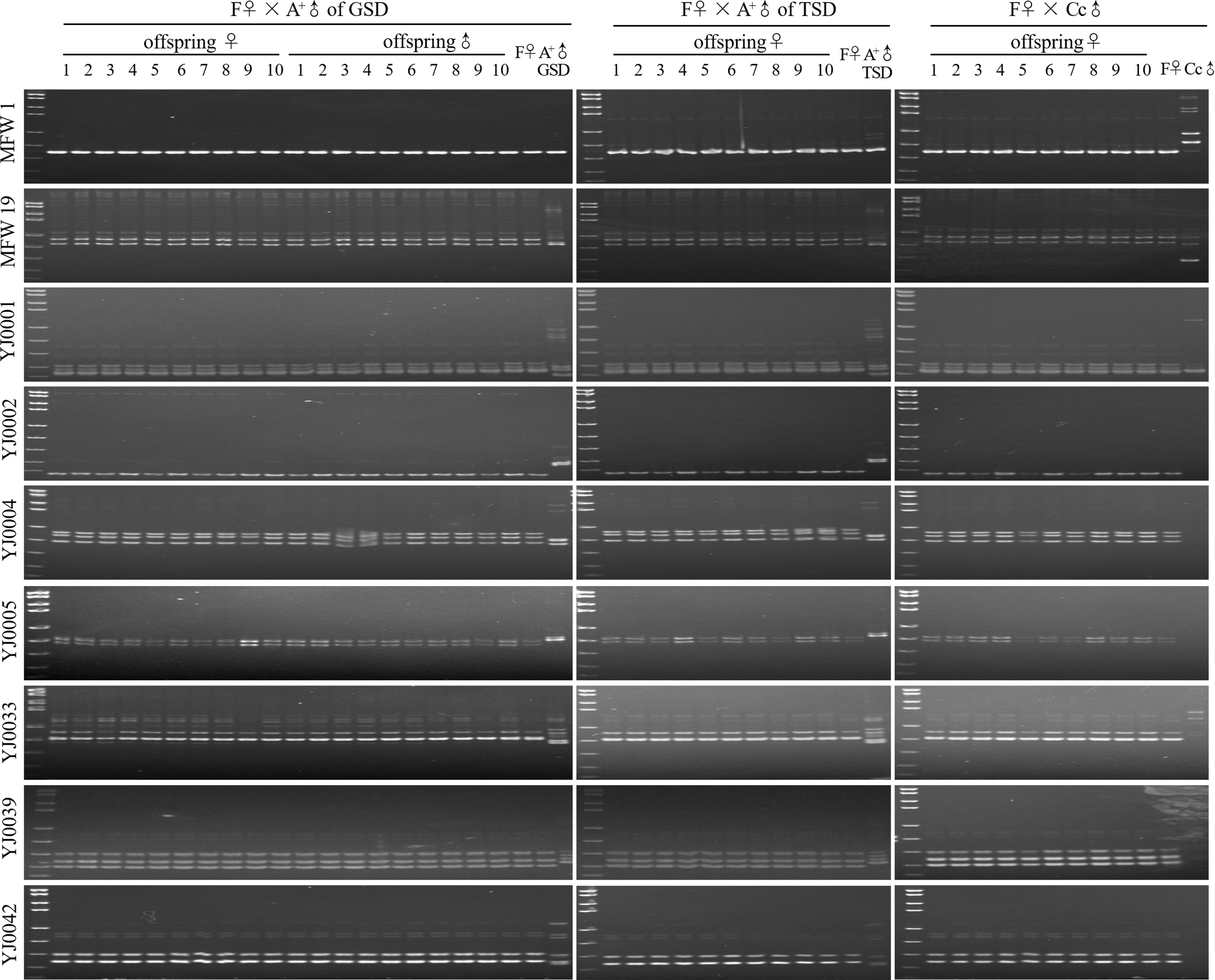

Supplement: Supplementary Figure 2 — Microsatellite analysis in the families of F♀ mating with A+♂ of GSD, A+♂ of TSD, and Cc♂. Microsatellite electrophoretic patterns amplified by the primer MFW1, MFW19, YJ0001, YJ0002, YJ0004, YJ0005, YJ0033, YJ0039, and YJ0042. ♀: female; ♂: male. Marker is pUC18 DNA/MspI. [file Image_2.tif]

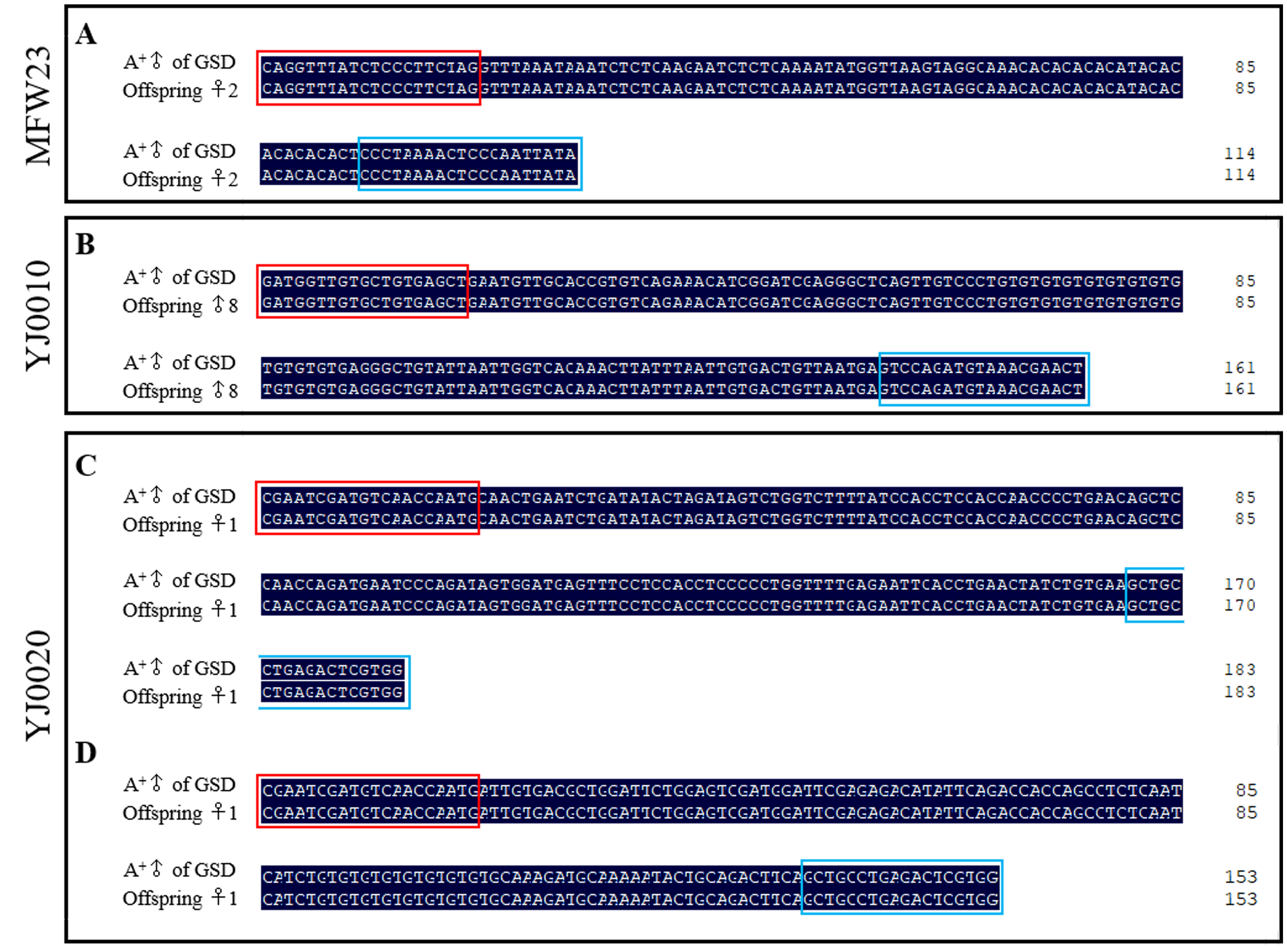

Supplement: Supplementary Figure 3 — Sequence alignments of the electrophoretic bands with the same size in both paternal A+♂ of GSD and corresponding offspring in Figure 3A. (A,B) Alignment of sequences amplified by primer MFW23 (A) and primer YJ0010 (B) between paternal individual A+♂ of GSD and the corresponding individual in the offspring. (C,D) Alignment of long sequences (C) and short sequences (D) amplified by primer YJ0020 between the paternal individual A+♂ of GSD and the corresponding individual in the offspring. Primers were exhibited on the left side. The forward and reverse primer sequences are indicated in red and blue rectangles, respectively. [file Image_3.tif]
