# Supplementary material for: Genotypic Males Play an Important Role in the Creation of Genetic Diversity in Gynogenetic Gibel Carp
Source: Front Genet. 2021 May 28;12:691923. doi: 10.3389/fgene.2021.691923 (PMC8194356; doi:10.3389/fgene.2021.691923)
Supplement: Supplementary Table 1 — Primer sequences and annealing temperature of genotypic male-specific marker. [file Table_1.DOCX]

**Supplementary Table S1** Primer sequences and annealing temperature of genotypic male-specific marker.

| Primers | Primer pair sequence (5’-3’) | T_a_ (℃) |
| --- | --- | --- |
| *Cg*-MSM | F: GCCACACTCACTTCTGTCTACA  R: ACTGCCATCTAACTCAGCCC | 58 |

T_a_: Annealing temperature.
