# Supplementary material for: Genotypic Males Play an Important Role in the Creation of Genetic Diversity in Gynogenetic Gibel Carp
Source: Front Genet. 2021 May 28;12:691923. doi: 10.3389/fgene.2021.691923 (PMC8194356; doi:10.3389/fgene.2021.691923)
Supplement: Supplementary Table 2 — Sequences and annealing temperatures of fifteen microsatellite primers. [file Table_2.DOCX]

**Supplementary Table S2**  Sequences and annealing temperatures of fifteen microsatellite primers.

| Microsatellite primers | Primer pair sequence (5'-3') | T_a_ (℃) |
| --- | --- | --- |
| MFW23 | F: CAGGTTTATCTCCCTTCTAG | 57 |
|  | R: GTATAATTGGGAGTTTTAGGG |  |
| YJ0010 | F:GATGGTTGTGCTGTGAGCT | 53 |
|  | R:GAGTTCGTTTACATCTGGAC |  |
| YJ0020 | F:CGAATCGATGTCAACCAATG | 50 |
|  | R:TCCACGAGTCTCAGGCAGC |  |
| YJ0022 | F:CACCAACTTTAGGCACATTTG | 53 |
|  | R:CCAGACTCCCACGTCATG |  |
| YJ0025 | F:GAGAAAGACCAGAATACAG | 50 |
|  | R:GGGAGACATTCATTTTATTG |  |
| YJ0040 | F:CCAGTATTAGGGAGCGTTC | 50 |
|  | R:GTTTCGTCTTCACAATCAGAA |  |
| MFW1 | F:GTCCAGACTGTCATCAGGAG | 58 |
|  | R:GAGGTGTACACTGAGTCACGC |  |
| MFW19 | F:GAATCCTCCATCATGCAAAC | 58 |
|  | R:GCACAAACTCCACATTGTGCC |  |
| YJ0001 | F:CTGGCATGAAGACTGGCTC | 53 |
|  | R:CAACAACACATATCAGCTCC |  |
| YJ0002 | F:CAAAAGCATGACAGAGTTAC | 50 |
|  | R:TGAAGTTATTAGAAAGAGAG |  |
| YJ0004 | F:CATAGAGGCGTTTCATAGAG | 50 |
|  | R:CAGATAAATACAGTAAGCCA |  |
| YJ0005 | F:TAATAAGGTACATAGTCATAG | 50 |
|  | R:GTCAGCCTCCACCACGAATC |  |
| YJ0033 | F:CGGACACAAGAACGCCAAC | 50 |
|  | R:GGACTGGGCTGAAACTGATG |  |
| YJ0039 | F:GAAGAATACTTTATGACTGAGG | 50 |
|  | R:GACCAAGACAGACAGCCCAG |  |
| YJ0042 | F:GGCCACCTACAGTATATGC | 50 |
|  | R:GAAAACCAGGACCGACATG |  |

T_a_: Annealing temperature.
